# Biosynthesis of Gold Nanoisotrops Using *Carallia brachiata* Leaf Extract and Their Catalytic Application in the Reduction of 4-Nitrophenol

**DOI:** 10.3389/fchem.2021.800145

**Published:** 2022-01-21

**Authors:** Najwa Ahmad Kuthi, Sheela Chandren, Norazah Basar, Mohamad Shazwan Shah Jamil

**Affiliations:** ^1^ Department of Chemistry, Faculty of Science, Universiti Teknologi Malaysia, Johor, Malaysia; ^2^ Centre for Sustainable Nanomaterials, Ibnu Sina Institute for Scientific and Industrial Research, Universiti Teknologi Malaysia, Johor, Malaysia

**Keywords:** biosynthesis, Carallia brachiata, gold nanoparticles (AuNPs), anisotropic, isotropic, 4-nitrophenol

## Abstract

The past decade has observed a significant surge in efforts to discover biological systems for the fabrication of metal nanoparticles. Among these methods, plant-mediated synthesis has garnered sizeable attention due to its rapid, cost-effective, environmentally benign single-step procedure. This study explores a step-wise, room-temperature protocol for the synthesis of gold nanoparticles (AuNPs) using *Carallia brachiata*, a mangrove species from the west coast of Peninsular Malaysia. The effects of various reaction parameters, such as incubation time, metal ion concentration, amount of extract and pH, on the formation of stable colloids were monitored using UV-visible (UV-Vis) absorption spectrophotometry. Our findings revealed that the physicochemical properties of the AuNPs were significantly dependent on the pH. Changing the pH of the plant extract from acidic to basic appears to have resulted in a blue-shift in the main characteristic feature of the surface plasmon resonance (SPR) band, from 535 to 511 nm. The high-resolution-transmission electron microscopy (HR-TEM) and field emission scanning electron microscopy (FESEM) images revealed the morphologies of the AuNPs synthesized at the inherent pH, varying from isodiametric spheres to exotic polygons and prisms, with sizes ranging from 10 to 120 nm. Contrarily, an optimum pH of 10 generated primarily spherical-shaped AuNPs with narrower size distribution (8–13 nm). The X-ray diffraction (XRD) analysis verified the formation of AuNPs as the diffraction patterns matched well with the standard value of a face-centered cubic (FCC) Au lattice structure. The Fourier-transform infrared (FTIR) spectra suggested that different functional groups are involved in the biosynthetic process, while the phytochemical test revealed a clear role of the phenolic compounds. The reduction of 4-nitrophenol (4-NP) was selected as the model reaction for evaluating the catalytic performance of the green-synthesized AuNPs. The catalytic activity of the small, isotropic AuNPs prepared using basic aqueous extract was more effective than the nanoanisotrops, with more than 90% of 4-NP conversion achieved in under an hour with just 3 mg of the nanocatalyst.

## 1 Introduction

Metal nanoparticles (NPs) have been adopted in various scholarly fields, medical and environmental research included. This is attributable to the novelty of their biophysical properties, which are primarily derived from their highly accessible surface. Gold nanoparticles (AuNPs), in particular, have caught the attention among various noble metals present and are among the nanometals commonly incorporated in biomedical science (Hu et al., 2020), such as for catalysis (Hutchings and Edwards, 2012) and sensors (Saha et al., 2012; Zhang, 2013). AuNPs boast of embedded physical and chemical attributes resulting in critical applications, which are tunable via size and shape modifications. Such processes are a key function for big-scale advanced material manufacturing, rendering an excellently regulated synthesis of the particles a necessity. This will straightforwardly ensure the correlation between NP’s structural and catalytic attributes (Walsh et al., 2012). Consequently, calls for practical, cost-effective, and environmentally safe processes have been made, as well as parameters favoring shape-regulated NP formation. In general, the nucleation and growth conditions of NPs are primarily impacted by parameters such as incubation time, metal ion concentration, pH, nature of reducing and stabilizing agents, seeds, and more. Eventually, these elements determine the final shape and geometry of the AuNPs formed (Shafey, 2020).

Conventional AuNP synthesis denotes the incorporation of chemical reducing agents. This practice emerges as significant over time due to its general toxicity yielding a multitude of biological side effects, thereby sparking doubts pertaining to the development of environmentally-friendly nanosynthesis routes (Mohanpuria et al., 2008; Sardar et al., 2009). Such chemical approaches are now outperformed by green NPs manufacturing by implementing plant-extracted biomolecules. Phyto-mediated NPs boast of heavily-regulated assembly attributable primarily to the dual functionalities of the extract as a reducing and capping agent. (Iravani, 2011; Akhtar et al., 2013). AuNP biosynthesis is well-known to be environmentally safe, cost-effective, and fit for mass manufacturing; it ensures excellent regulation of the particle size distribution across different manners (Ahmed et al., 2016). The straight-forward and safe-for-environment properties of such biosynthetic approaches that implement plant extracts have sparked interest in recent years (Castillo-Henríquez et al., 2020; ElMitwalli et al., 2020; Botteon et al., 2021).

*Carallia brachiata* (Lour.) Merr. of the family Rhizophoraceae presents a medium-sized tree typically found in lowlands (i.e., swamps) and seldomly in hill forests. It is known as Meransi in Malaysia and generates hard timbers for furniture manufacturing and interior finishing (Hou, 1957). Alternatively, the plant’s leaves are incorporated in traditional medicinal tea and commonly used as sapremia treatment in combination with turmeric, benzoin, and rice dust (Burkhill et al., 1966), whereas its bark can be employed for treating itching, cuts, and wounds (Sastri, 1962). Among the pioneering phytochemical works undertaken in the 1960s, one has particularly described the presence of (+)-hygroline as a major alkaloid in the leaves (Fittzgerald, 1965). Decades later, researchers were finally able to isolate and identify a new diglycosyl megastigmane from the species, delineating 29 additional secondary metabolites, such as megastigmanes, 1,2-dithiolane derivative, phenolic compounds, condensed tannins, flavonoids, and glyceroglycolipids (Ling et al., 2004). Abundant secondary metabolites in mangroves and other plants found in the ecosystem notwithstanding, the least amount of works can be found to ascertain the possibility of their role as a source for biogenic NP synthesis.

Bulk Au has long been perceived as a nondescript metal in terms of its catalysis. Contrarily, its catalytic attributes are particularly evident upon a size reduction to mere nanometers, depicting a highly catalytic activity toward a variety of chemical reactions (Sankar et al., 2020). In particular, such activity held by biosynthesized AuNPs is typically assessed via 4-nitrophenol (4-NP) reduction. In the context of manufactured quantity and extent of environmental contamination, 4-NP is a typical and critical industrial waste, well-known to be a chemically resistant and non-biodegradable pollutant (Uberoi and Bhattacharya, 1997). Its release into marine reservoirs elevates its status as the primary constituent of poorly-treated industrial effluent and thus frequently identified in bodies of water. Such findings undoubtedly affect the aquatic environment directly while humans are influenced indirectly. The combination of its non-biodegradable status and such hazardous implications to humans and the environment alike has rendered 4-NP registered on the United States Environmental Protection Agency’s Pollutant List (Tchieno and Tonle, 2018; Yahya et al., 2021). This list names it as a priority pollutant and calls for instantaneous environmental remediation. Consequently, the conversion of simple or complex 4-NP to its non-hazardous intermediate, 4-aminophenol (4-AP), is deemed critical for eradicating environmental contamination generated due to the chemical. In this case, the abundance and diversity offered by nanocatalytic systems via reducing agent utilization have resulted in the evolution of substrates possibly equipped for industrially-useful reduction of 4-NP (Mehmood et al., 2016; Rodríguez Molina et al., 2019).

This work pioneers the design of a single-step room-temperature AuNP synthesis via *C. brachiata* incorporation, which is a mangrove species commonly found on the west coast of Peninsular Malaysia. It implements a fully green procedural, completely bypassing the use of any toxic chemicals in the synthesis protocol. Furthermore, NPs size, shape, and morphological properties are modulated and enhanced by controlling the reaction conditions, such as incubation time, precursor metal ion concentration, extract quantity, and pH. Concurrently, the present work describes the catalytic potential of the enhanced AuNPs for the purpose of 4-NP degradation using hydrazine hydrate, thus paving the way for new possibilities in future bioremediation programs.

## 2 Experimental

### 2.1 Materials

Tetrachloroauric (III) acid trihydrate, HAuCl_4_.3H_2_O (99.9%) and 4-NP were purchased from Sigma-Aldrich and were used without further purification. Hydrochloric acid (HCl), concentrated sulphuric acid (H_2_SO_4_), sodium hydroxide pellets (NaOH), ferric chloride hexahydrate (FeCl_3_.6H_2_O) and Mayer’s reagent were acquired from Merck (M) Sdn. Bhd. while hydrazine monohydrate was obtained from Honeywell Fluka. Deionized water (MQ-H_2_O) was used throughout the experiments. Fresh *C. brachiata* leaves (H051) were collected from Ayer Keroh Forest Reserve, Malaysia. The specimen was identified by Dr. Mohd Nazre Salleh and deposited at the Faculty of Forestry, Universiti Putra Malaysia.

### 2.2 Preparation of *C. brachiata* Aqueous Extract

The *C. brachiata* leaves were thoroughly washed with deionized water to remove mud, dust particles and other foreign matter and dried at room temperature under shade for 1 week. The resulting dried plant material was then ground to powder state in a vertical pulverizing machine before it was soaked in deionized water and subjected to heat on a stirring hotplate at 60°C for 30 min. Next, filtration was performed on the mixture to remove any leftover biomass, while the filtrate obtained was kept in an airtight amber glass vial and refrigerated at 5°C until analytical processes commenced. This work specifically assessed the potential effects of pH on AuNP biosynthesis by subjecting the prepared aqueous extract to acidic and alkaline treatments with diluted HCl and NaOH solutions, respectively.

### 2.3 Phytochemical Screening of the *C. brachiata* Extract

A stock concentration of 10% w/v extract was prepared using deionized water. The extract was analyzed qualitatively to detect the presence of phenolic compounds, flavonoids, terpenoids and alkaloids following standard methods (Kwan et al., 2013);

#### 2.1.1 Detection of Phenolic Compounds

To the prepared sample, few drops of 5% w/v FeCl_3_.6H_2_O solution were added. The formation of bluish-black color indicates the presence of phenols.

#### 2.1.2 Detection of Flavonoids

The prepared sample was first treated with 2% w/v NaOH solution, followed by a few drops of HCl (2M). The formation of vibrant yellow color followed by decoloration upon acid treatment indicates the presence of flavonoids.

#### 2.1.3 Detection of Terpenoids

A small amount of lyophilized extract was shaken in 1 ml of chloroform prior to adding a few drops of concentrated H_2_SO_4_. A red brown color formation at the interface confirmed the test.

#### 2.1.4 Detection of Alkaloids

Two mL of Mayer’s reagent was added to 1 ml of the prepared sample. The formation of red precipitate indicates the presence of alkaloids.

### 2.4 Synthesis of AuNPs

Biosynthesis of AuNPs involved the mixing of aliquot amounts of HAuCl_4_ and *C. brachiata* extract. A sufficient amount of *C. brachiata* extract was subjected to a reaction with 1 mM HAuCl_4_ solution in a scintillating vial and at room temperature under constant motion. AuNP formation was confirmed via color shifts from pale yellow to stable violet, and the resulting colloidal solution was centrifuged at 13,000 rpm for 15 min. Then, it was re-dispersed in deionized water several times to completely remove any free entities. The resulting suspension was dried in a vacuum desiccator prior to analysis.

### 2.5 Characterization of the Synthesized AuNPs

The bio-reduction of the [AuCl_4_]^−^ ions in the solution was monitored by measuring the UV–Vis spectra of the colloidal solution in quartz cuvettes with a double-beam spectrophotometer (Shimadzu UV-1800) in the range of 400–800 nm. The morphology of the nanoparticles was analyzed using the images obtained with a JEOL ARM 200 F TEM. Alternatively, AuNP characterization was performed using a Hitachi SU8020 SEM instrument equipped with an XmaxN EDAX attachment. The FT-IR spectra were recorded on a Frontier Perkin-Elmer FTIR instrument outfitted with a diamond attenuated total reflectance (ATR) accessory. Meanwhile, the XRD patterns of dried nanoparticle pellets were generated using Panalytical X'PertPro diffractometer with monochromatized CuKα radiation (l = 1.5406 Å). The average crystallite size of the synthesized AuNPs was calculated using Scherrer’s formula:

$$D=\frac{K\lambda }{\left(\beta \cos\theta \right)}$$ (1)

where D is the average crystallite size, K is the Scherrer’s constant, *λ* is the X-ray wavelength, β is the full-width at half maximum (FWHM), and θ is the Bragg’s angle (i.e., displayed in degrees).

### 2.6 Catalytic Activities of the Biosynthesized AuNPs in the Reduction of 4-NP

In this study, the 4-nitrophenol (4-NP) reduction reaction was the chosen model employed in assessing the catalytic performance of green-synthesized AuNPs. Here, equal parts of freshly prepared 4-NP (0.1 mM) and hydrazine hydrate (0.3 mM) were subjected to constant mixing at room temperature. The mixture was supplemented with 1 mg of AuNP pellets, following which monitoring of the reduction reaction process was completed using a UV-vis spectrophotometer and recorded in 5- or 10-min intervals. The same procedures were repeated using the addition of 2, 3, and 4 mg of AuNP pellets to the point of reduction completion. The catalytic degradation efficiency (D %) of the biosynthesized AuNPs was calculated using the following formula:

$$D=\;\frac{\left(A_{0}-A_{t}\right)}{A_{0}}\times 100\%$$ (2)

where A_t_ and A_0_ are the absorbances of 4-NP at 400 nm at a particular time interval and at time 0, respectively.

## 3 Results and Discussion

### 3.1 Phytochemical Screening Tests

Preliminary phytochemical screening was carried out using established procedures outlined by Kwan et al. (2013) to identify the presence of phyto-constituents in the aqueous extract. It involves observing the visual color change caused by different reagent treatments, each of which indicates a different class of phytochemical. In general, these qualitative tests shed light on the nature of secondary metabolites present in the *C. brachiata* leaf extract.

Table 1 depicts the outcomes of qualitative screening subjected to the phytochemical components of aqueous *C. brachiata* leaf extract. The findings revealed a high concentration of phenolics and a trace amount of flavonoids, but neither alkaloids nor terpenoids were recorded. Both phenolics and flavonoids are powerful antioxidants and bio-capping agents. They are capable of improving NP stability, preventing agglomeration and deformation and increasing the reaction rate of NPs *via* surface adsorption (Shafey, 2020).

**TABLE 1:** Preliminary phytochemical screening of aqueous *C. brachiata* leaf extract.

Constituents	Results
Phenolic Compounds	+++
Flavonoids	+
Terpenoids	−
Alkaloids	−

*+ = Trace, ++ = Moderate, +++ = Abundant, − = Absent

### 3.2 UV-Visible Analyses

#### 3.2.1 Effect of Incubation Time

**FIGURE 1:**
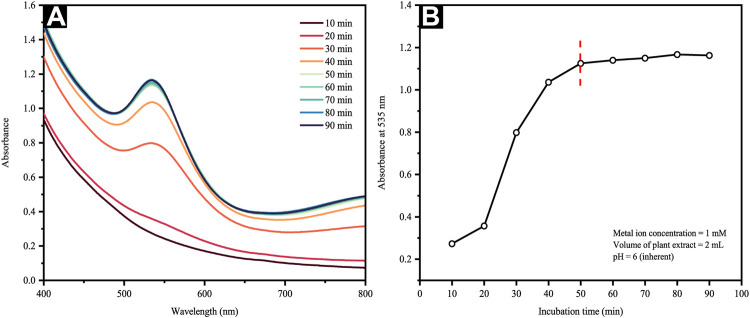
**(A)** UV-Vis spectra of the reaction mixture at different time intervals and **(B)** change in absorbance of Au colloidal solution at 535 nm as a function of time.

UV-vis spectroscopy was employed to assess AuNP formation and particle size shifts seen in an aqueous solution. The absorption peak was designated to the surface plasmon resonance (SPR) band of AuNPs following the collective conduction electron oscillation of the NPs, which were instigated by the incident light field. The SPR would depict an intensity and width dependent upon the particle size, morphology, spatial orientation, optical constants and the embedding medium utilized (Ashkarran and Bayat, 2013). Figure 1A reveals the UV–Vis spectra of AuCl4− solution following the addition of extract (2 ml) as a function of time. They showed a rapidly increasing absorption peak at about 535 nm within the first 50 min, which was followed by an extremely slow absorption increment. An assessment of AuNP formation kinetics was then performed by recording any changes in the band absorbance at *λ* = 535 nm with respect to time. The outcomes are shown in Figure 1B, in which the saturation is attained within 50 min of reaction in the reduction process, following which minimal variation is seen in the band intensity.

#### 3.2.2 Effect of Metal Ion Concentration

**FIGURE 2:**
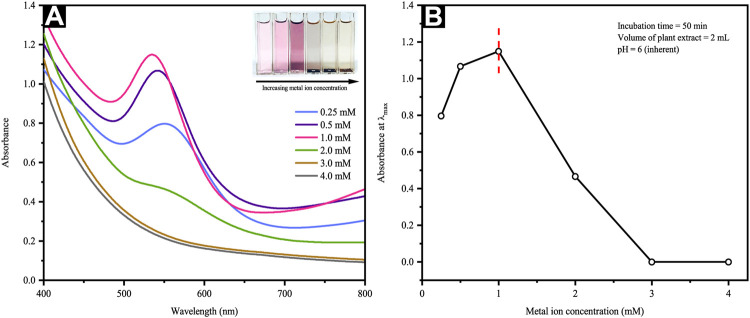
**(A)** UV-Vis spectra and optical images of reaction mixtures at different metal ion concentrations and **(B)** a plot of maximum absorbance versus precursor metal ion concentration.

The LaMer model projects that NP formation is only possible should the precursor concentration used is within the range suited for nucleation (Thanh et al., 2014). Nevertheless, such a range may be modified in view of various biomass-aided synthesis approaches present. Thus, this work ascertained the effect of precursor concentration on CB-mediated synthesis. Figure 2A reveals the SPR bands, which vary from 535–556 nm and rely upon the metal precursor concentration. An increment of metal precursor concentration from 0.25 to 1 mM further resulted in a steady absorbance increase, whereby the representative SPR band (i.e., 535 nm) was detectable at 1 mM. Contrarily, the lack of further SPR band absorbance increments beyond 1 mM was indicative of phyto-synthetic saturation (Figure 2B). Above 1 mM of the Au gold precursor salt concentrations, the Au ions revealed a rapid reduction, aggregation, and precipitation, whereby the precipitation was particularly visible to the naked eye (inset of Figure 2A). Consequently, the subsequent experiments were carried out at a fixed gold salt concentration of 1 mM. Such results echo those reported by Ahmad et al. (2017), which incorporated *Mentha piperita* leaf extract. In particular, the scholars employed higher metal precursor concentrations ranging from 1 to 5 mM, correlating the NP aggregation increment with the HAuCl_4_ concentration. This is said to occur following the competition present between auric ions and the functional groups of the leaf extract.

#### 3.2.3 Effect of Extract Quantity

**FIGURE 3:**
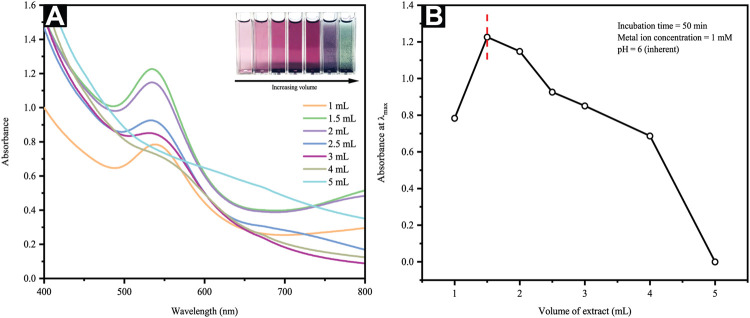
**(A)** UV-Vis spectra and optical images of reaction mixtures at different extract volumes and **(B)** a plot of maximum absorbance versus extract volume.

The quantity of plant extract used is a crucial parameter for controlling the phytosynthesis and predicting the extent of NP agglomeration. Figure 3A depicts the UV–Vis spectra of AuNP formation via constant HAuCl_4_ concentration (1 mM) and varying extract volumes, whereby the inset presents the photos of AuNP color changes with different CB extract volumes. An observation of violet pink to dark magenta shift was attributable to the SPR of different-sized AuNPs. In this study, the quantity of leaf broth extracts ranged from 1 to 5 ml in which the lower amounts (i.e., 1–1.5 ml in 10 ml metal ion solution) reflected a gradual increment of the absorption spectra (Figure 3B). Contrarily, higher extract volume added (i.e., 2–5 ml) depicts a shift to the λ_max_, namely longer wavelengths and green-colored AuNP solution, as seen in the inset of Figure 3A. Furthermore, a maximum absorption increment and negligible absorbance shift were seen at 1.5 ml extract volume, suggesting a saturated state in the bio-reduction of Au^3+^. Regardless, the absorption maximum at about 535 nm was clearly characteristic for the SPR band of the AuNPs formed. Moreover, light interaction of smaller wavelength that the AuNP particle size yielded the polarization of free conduction electrons in line with the latter’s heavier ionic core, thus creating a dipolar electron oscillation and generating an SPR band.

Following an increased reaction rate, consumption of most Au^3+^ ions occurred in the nuclei formation, thereby depicting smaller particle sizes. Such observation would imply the critical value of precursors (i.e., extract/Au^3+^ ratio) as a parameter influencing the nucleation and growth processes of AuNP formation. The decreasing particle size observed following increments of extract volume from 1 to 1.5 ml occurred as the absorption spectra were blue-shifted. On the contrary, an increment of extract volume from 2 to 5 ml, as seen in Figure 3A, yields slight red shift in λ_max_ concurrent with noticeable absorbance decrement (Figure 3B), agreed the prior outcomes for surfactant-free DNA-functionalized AuNPs (Li et al., 2017). These observations are highly possibly linked to the dielectric attribute changes of the layer immediately surrounding the AuNPs and a minimal amount of particle aggregation. Theoretically, the use of excessive reducing agents in amount is likely to trigger a secondary reduction process on the preformed nuclei surface, thus causing the formation of larger AuNPs. Furthermore, increasing the extract volume from 2 to 5 ml reveals an incrementally darkening color of the AuNP colloidal generated as per the inset of Figure 3A. Therefore, such outcomes substantiated the strong dependence of AuNP particle size and distribution on the C. *brachiata* leaf extract to Au^3+^ ions concentration ratio.

#### 3.2.4 Effect of Extract pH

**FIGURE 4:**
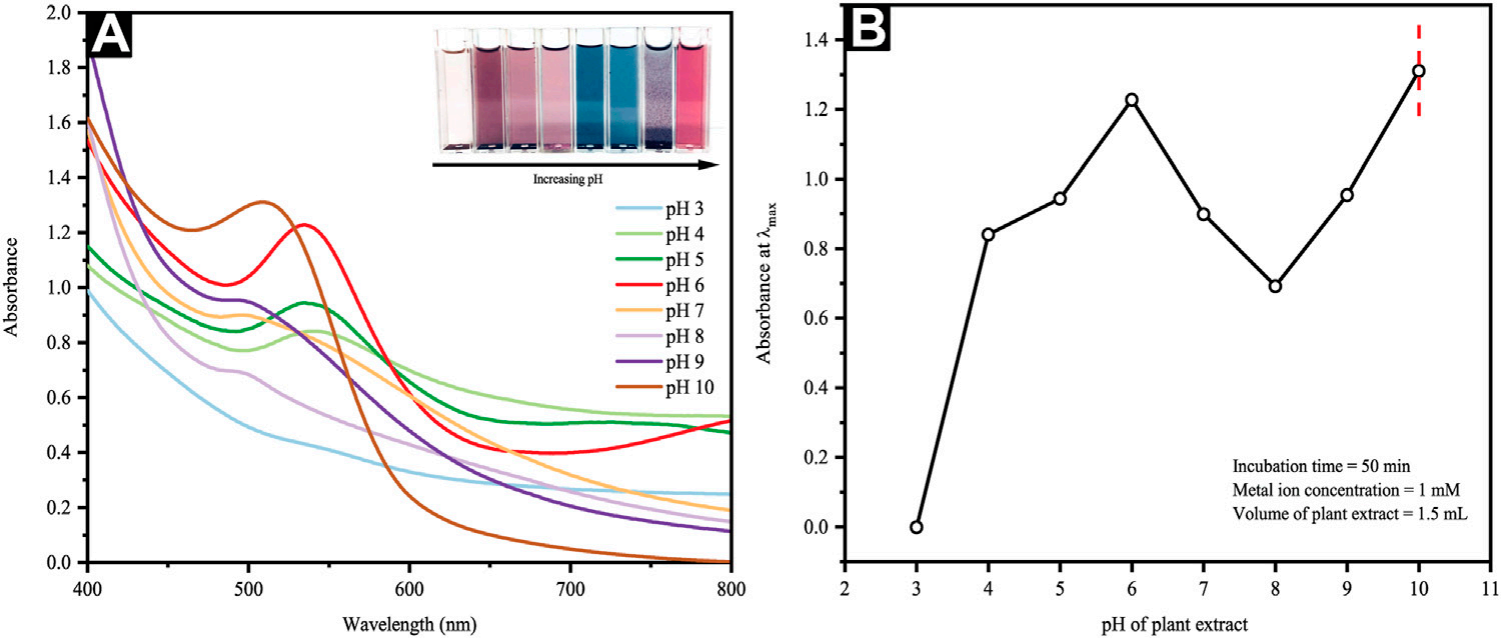
**(A)** UV-Vis spectra and optical images of reaction mixtures at different pH and **(B)** a plot of maximum absorbance versus extract pH.

AuNP synthesis *via* CB incorporation underlined the critical function of the initial pH value of the aqueous extract solution in which the nano Au solutions changed color from nepheloid magenta to greyish blue and subsequently to coral pink upon increments of initial pH (i.e., from 6 to 10). This corresponded with their UV-vis spectra at different pH values, whereby the SPR bands of AuNPs were located at various positions at dissimilar pH values. Concurrent with the incremental pH values, the blue shift of the peak position was consistently observed in the UV-vis spectra and moved alongside the decremental AuNP sizes (Figure 4A).

Au typically occurs in its anionic form of [AuCl_4_]^-^ in an acidic solution; here, the functional groups of CB and hydroxyl are likely to go through protonation and become positively charged. Such a positively charged surface would then aid the interactions of protonated hydroxyl groups and negatively charged [AuCl_4_]^-^ via electrostatic attraction or an electrovalent bond (Zhou et al., 2010). Consequently, a preference for biosorption can be seen as opposed to the bio-reduction of [AuCl_4_]^-^. The growth process generally leads to elongated Au seeds rather than new Au nuclei formation, thus yielding anisotropic AuNPs of larger dimensions (Xiao et al., 2004; Zayats et al., 2005; Willner et al., 2006; Gajanan et al., 2011). The increasing pH value shows a significant reduction of absorbance at pH 7 and 8, as seen in Figure 4B, whereas a pH value of 10 renders OH^−^ a strong complexing agent of Au ions. They would disrupt the CB capping capacity and compete with [AuCl_4_]^-^ for biomolecular binding. The resulting AuNPs are primarily spherical, and the near disappearance of anisotropic AuNPs. Such [AuCl_4_]^-^ bio-reduction emerges following hydroxyl oxidation, yielding carbonyl groups, as shown in the equation below:[HAuCl_4_]^-^ + 3R-OH_n_ → Au^0^ + 3 R=O + 3nH^+^ + 4Cl^-^where n is the number of hydroxyl groups in the biomolecules.

The reaction stoichiometry revealed the inclusion of three electrons and three protons transferred during the redox reaction, indicating the possible pH dependency of the kinetics and standard redox potentials. Furthermore, an alkaline environment resulted in the rapid reduction rate of [AuCl_4_]^-^, improved homogenous nucleation, and reduced anisotropic growth. Contrarily, a slow reduction rate would be expected in acidic environments, yielding heterogeneous nucleation and secondary nucleation of small Au seeds.

### 3.3 Possible Mechanism for the Formation and Complexation of AuNPs by *C. brachiata*

**FIGURE 5:**
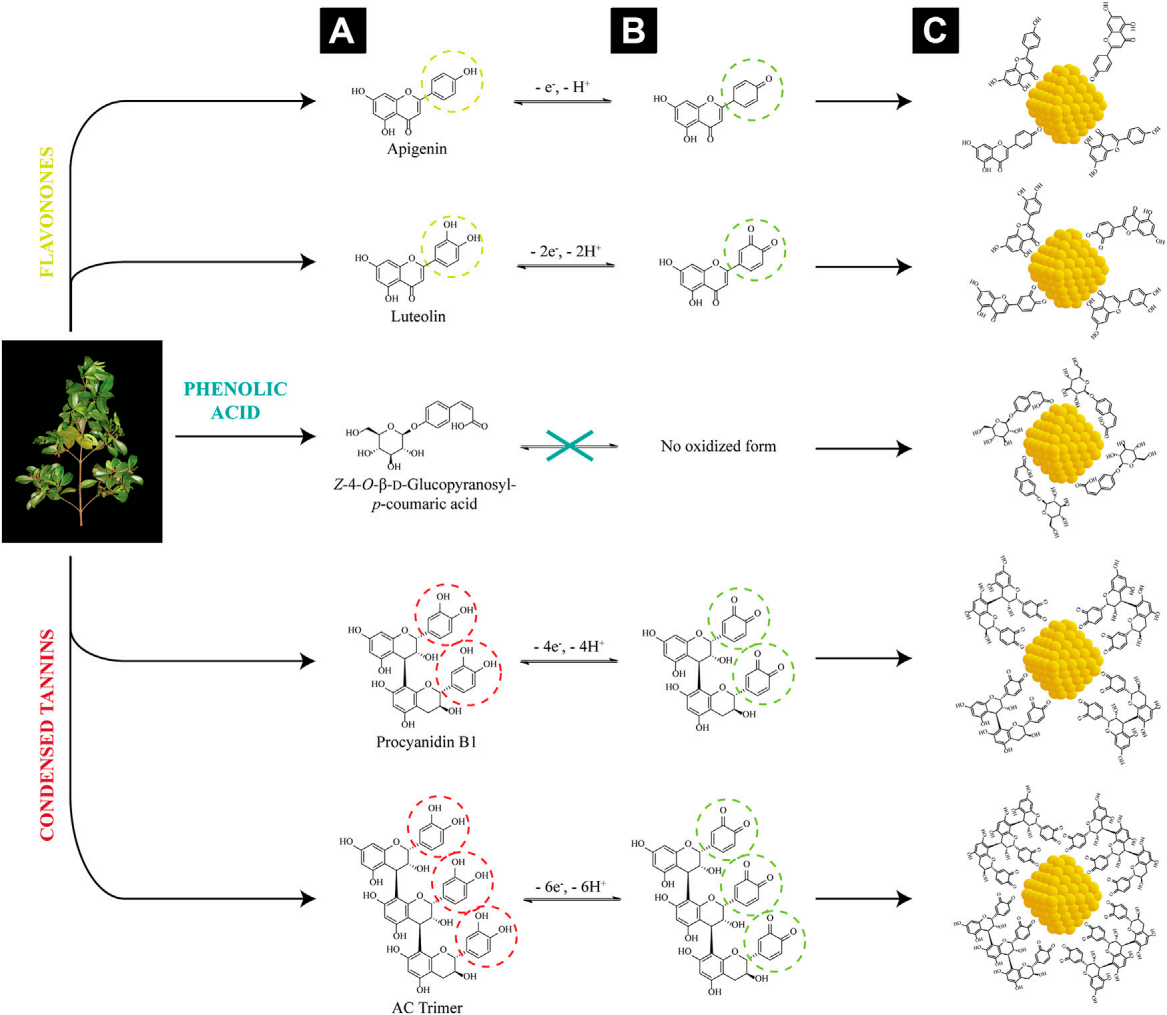
Proposed mechanism for the reduction and stabilization of AuNPs by polyphenolic compounds of *C. brachiata*: **(A)** previously reported phenolics and flavonoids from the leaf extract of *C. brachiata*, **(B)** their corresponding oxidized forms, and **(C)** and their complexation on the AuNP surfaces.

The work by Ling et al. (2004) has described the multitude of medicinal compounds from the dried leaf extract of *C.* brachiata, namely 3-hydroxy-5,6-epoxy-β-ionol-3-*O*-β-apifuranosyl-(1→6)-β-glucopyranoside, corchoionoside A, vormifoliol, Z-4-*O*-β-D-glucopyrasonyl-*p*-coumaric acid, luteolin, apigenin, procyanidin B1 and AC trimer. Preceding phytochemical tests had merely insinuated that phenolics and flavonoids are present in the aqueous extract, suggesting the role of five phytochemicals (Figure 5A) in the reduction and complexation of AuNPs. Generally, the reduction of Au^3+^ ions to Au^0^ occurs concurrently with the oxidation of the hydroxyl groups to quinones (Figure 5B). The collision between neighboring Au^0^ atoms forms AuNPs, which are then stabilized by the polyphenolic compounds, their corresponding oxidized forms (Figure 5C), as well as other coordinating metabolites present in the extract.

Apigenin and luteolin, both flavones from the flavonoid family, are susceptible to oxidation. The hydroxyl group(s) in their phenolic ring B heavily matter in the process of hydrogen transfer reactions since it is the only structural difference between them (Panche et al., 2016; Wang et al., 2018). The mechanism of electrochemical oxidation of the flavonoids, particularly of 3′,4′-catechol group has been reported multiple times (Ghica and Brett, 2005; Timbola et al., 2006; Liu et al., 2008; Ramešová et al., 2013). The oxidation of the catechol moiety which involves the reversible transfer of two electrons and two protons results in the formation stable *ortho*-quinones *via* the resonance effect. Since apigenin lacks a 3′-hydroxy group, and it does not oxidize to form a quinoid product similar to that of luteolin (Mülazimolu and Solak, 2011). Eventually, the π bonds in their oxidized structures coordinate and chelate AuNPs, forming a steric layer on the particle surface to inhibit aggregation (Ahmad et al., 2019). The phenolic acid derivative, Z-4-*O*-β-D-glucopyrasonyl-*p*-coumaric acid, on the other hand, does not participate in the electron transfer reaction at all due to the absence of hydroxyl group in its structure considering the *para*-substitution for sugar moiety. However, the molecule can still be chemisorbed onto the AuNPs via its carboxylate group, with its two oxygen atoms symmetrically bound to the NP surface. This has also been observed with many other benzoic acid derivatives such as andanthraquione-2-carboxylic acid (Han et al., 2000), cinnamic acid (Wang et al., 2006), benzoic acid and phthalic acid (Gao et al., 2013). Procyanidin dimers and trimers like procyanidin B1 and AC trimer showed excellent potential as antioxidant agents due to their polyhydroxyl structures and ease of transferring electrons and protons to metals by the virtue of oxidation of their catechol forms in a similar manner to that of luteolin. A five-membered chelate ring can form between their *ortho-*quinone groups and the AuNPs (Biao et al., 2018; Badeggi et al., 2020a, 2020b).

### 3.4 Morphological Studies

**FIGURE 6:**
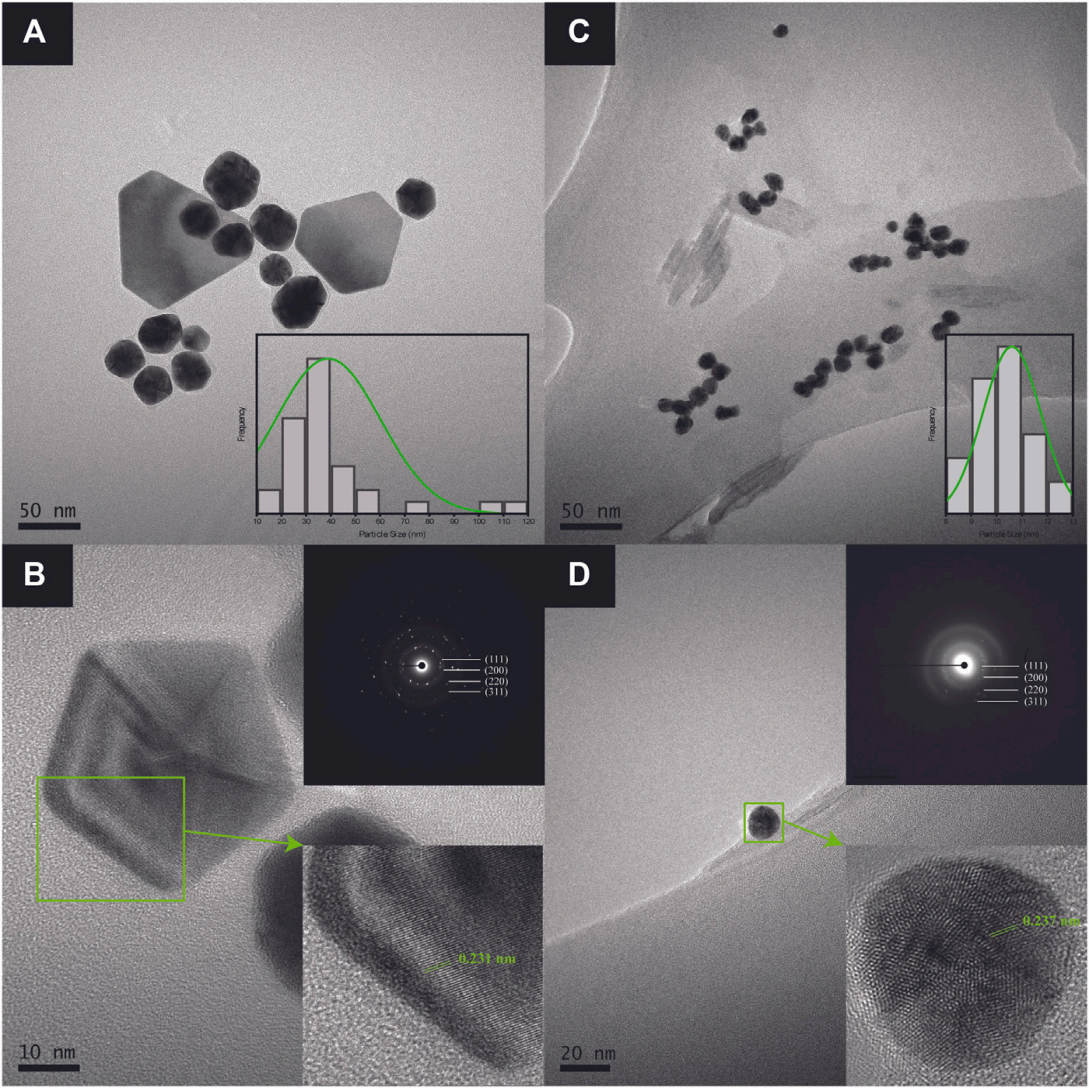
HR-TEM images of **(A)** anisotropic AuNPs@pH6 with corresponding particle size distribution and **(B)** a single pentagonal crystal structure with its d-spacing and electron diffraction pattern **(C)** isotropic AuNPs@pH10 with corresponding particle size distribution and **(D)** a single spherical crystal structure with its d-spacing and electron diffraction pattern.

**FIGURE 7:**
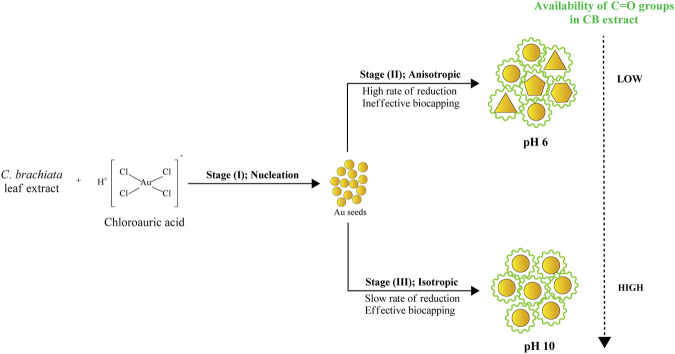
Morphological changes in AuNPs as a function of pH.

This study undertook HR-TEM and FESEM measurements to determine the morphology and particle size distribution of Au atoms biosynthesized with pH-6 and pH-10 extracts. The TEM results of AuNPs@pH6, in particular, depict primarily spherical-shaped Au particles formed and few traces of triangular and pentagonal particles as revealed in Figure 6A,B. Due to the thickness of larger anisotropic structure, the electron diffraction could only be collected from the edge of the particle. The lattice spacing of 0.231 nm corresponding to the (111) plane is clearly evident from the surface of Au nanopentagon. Futher, the bright, well-defined spots in the SAED patterns (inset of Figure 6B) have confirmed the polycrystalline nature of the biosynthesized AuNPs@pH6. Meanwhile, Figure 6C shows partially agglomerated spherical particles for AuNPs@pH10 with an average diameter of 10.56 ± 2.33 nm. The presence of a few distinct diffraction spots of low intensity observed for single spherical AuNPs@pH10 (inset of Figure 6D) may be attributed to their small crystallite size (Sharma and Deswal, 2018). Here, the AuNP morphology was predominantly affected by the extract’s pH, in which its morphological behavior shifted following an increment of pH. pH spanning from 6 to 10 are thus depicted in Figure 7 using three suggested stages, namely: (I) Nucleation, (II) Anisotropic, and (III) Isotropic.

**FIGURE 8:**
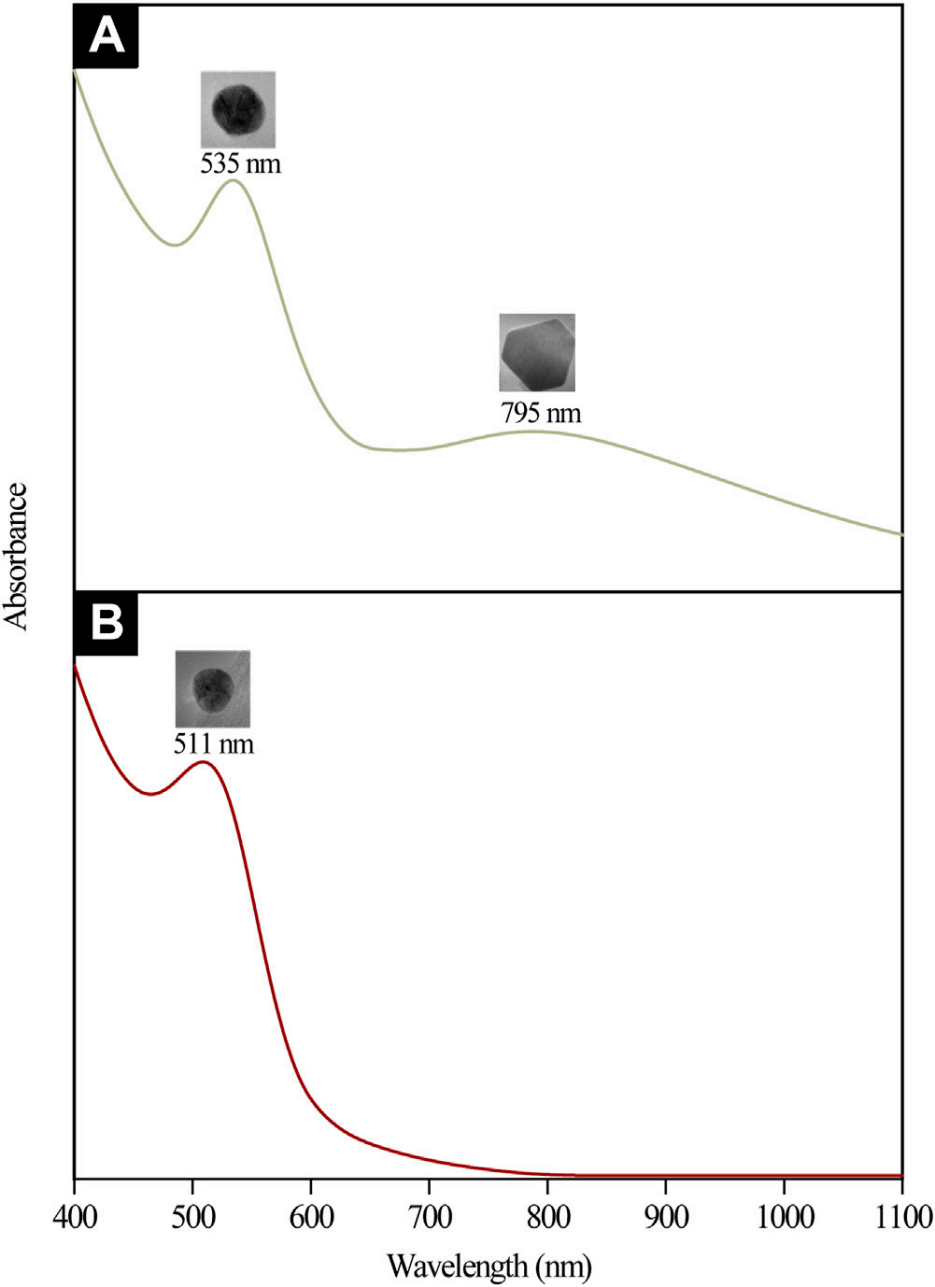
UV-Vis-NIR absorption spectra and the corresponding TEM micrographs of **(A)** anisotropic AuNPs@pH6 and **(B)** isotropic AuNPs@pH10

Stages (I) and (II) concurrently depict the nucleation and anisotropic processes forming the nuclei (seeds) in ensuring further catalyzation of crystal growth (Thanh et al., 2014). This yields the formation of triangular, pentagonal, spherical, and hexagonal-shaped AuNPs as displayed in Figure 6A,B. The bio-reduction reaction requires the bio-capping agents to control the NP size and morphology, which may embed on non-specifically open surfaces of AuNPs and cause the anisotropic morphological formation. Swami et al. (2018) have previously delineated the projected function of hydroxyl ions toward attaining the anisotropic morphology of AuNPs synthesized using hydroxyl-moiety-bearing amino acids. Thus, it was hypothesized that multiple-shaped AuNPs could be substantiated upon being subjected to varying pH, spanning from 3 to 10. The early stages of reaction rendered the bio-capping agents available in the reaction mixture incapable of causing an effectual stabilization of all AuNPs, which were rapidly formed due to high reduction reaction rates. In particular, the anisotropic stage was deemed a fast stage following ineffective phenolic, alcoholic, and amide functional group capping. Meanwhile, Stage (III) depicted a clear morphological shift, with nearly all Au seeds growing into predominantly spherical-shaped NPs. This was also evident from the absorption spectra of CB reduced/capped AuNPs (Figure 8), where a transverse located at 511 and 535 nm can be seen for both samples but only AuNPs@pH6 exhibited a weak longitudinal band in the near-IR region (700–1,000 nm). The appearance of this longitudinal band confirms the formation of anisotropic nanostructures (Sakai and Alexandridis, 2005).

**FIGURE 9:**
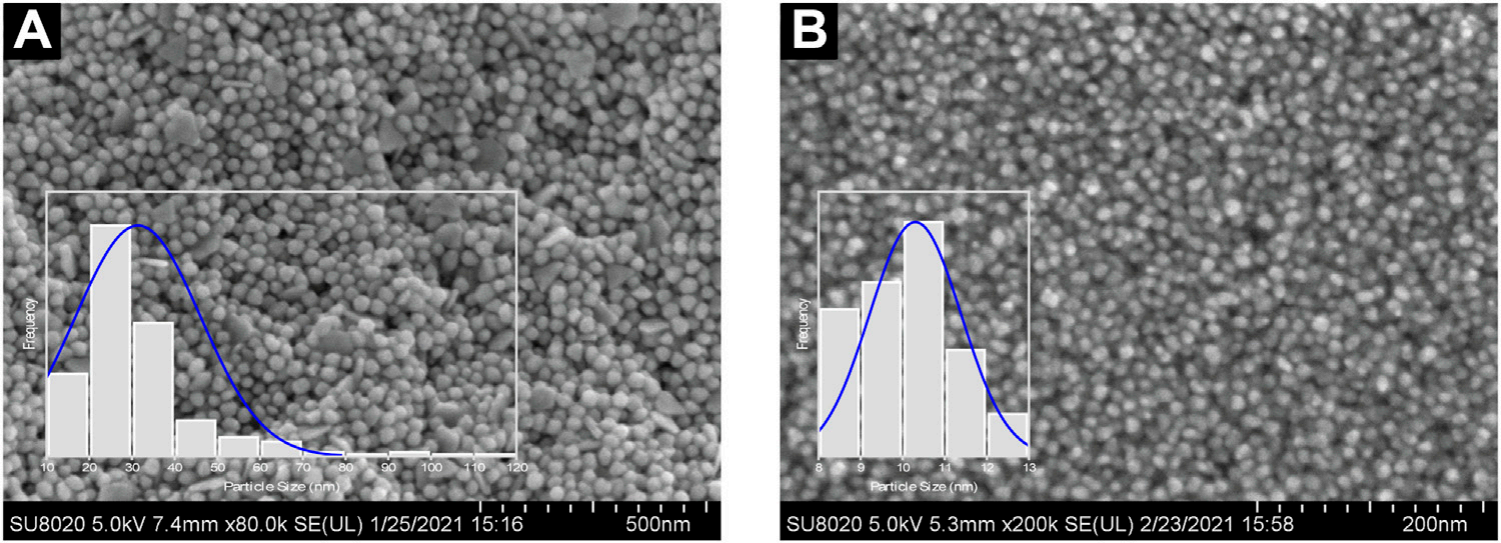
FESEM images and particle size distribution of **(A)** AuNPs@pH6 and **(B)** AuNPs@pH10

Following the anisotropic and isotropic stages, Ostwald’s Ripening mechanisms should occur. The isotropic stage, for example, was comparably slower than the anisotropic stage, given low reduction rates and less AuNP yield. Accordingly, the formation of monodispersed and isotropic AuNPs was attributable to slower reduction reaction and effectual newly-formed NP stabilization as a result of bio-capping agents prevalent in the reaction mixture. FESEM images revealed in Figure 9A,B further substantiate the significant importance of pH in ascertaining the NP morphology. AuNPs@pH10 displayed small spherical size with a narrow distribution (8–13 nm) while AuNPs@pH6 exhibited larger anisotropic nanocrytals ranging from 10 to 120 nm. The findings of this study corroborate those of Queslati et al. (2020). The scholars have identified a narrow particle size distribution for AuNPs upon testing with incremental pH. Here, the AuNP shape is critical in delineating their catalytic and biological properties. For instance, Benedec et al. (2018) and Goyal et al. (2019) have described multiple-shaped AuNPs biosynthesized using oregano and cinnamon extracts, respectively. The scholars have attributed the superior pharmacological activities of anisotropic AuNPs to their better biocompatibility (Ankamwar, 2012).

### 3.5 Elemental Analysis

**FIGURE 10:**
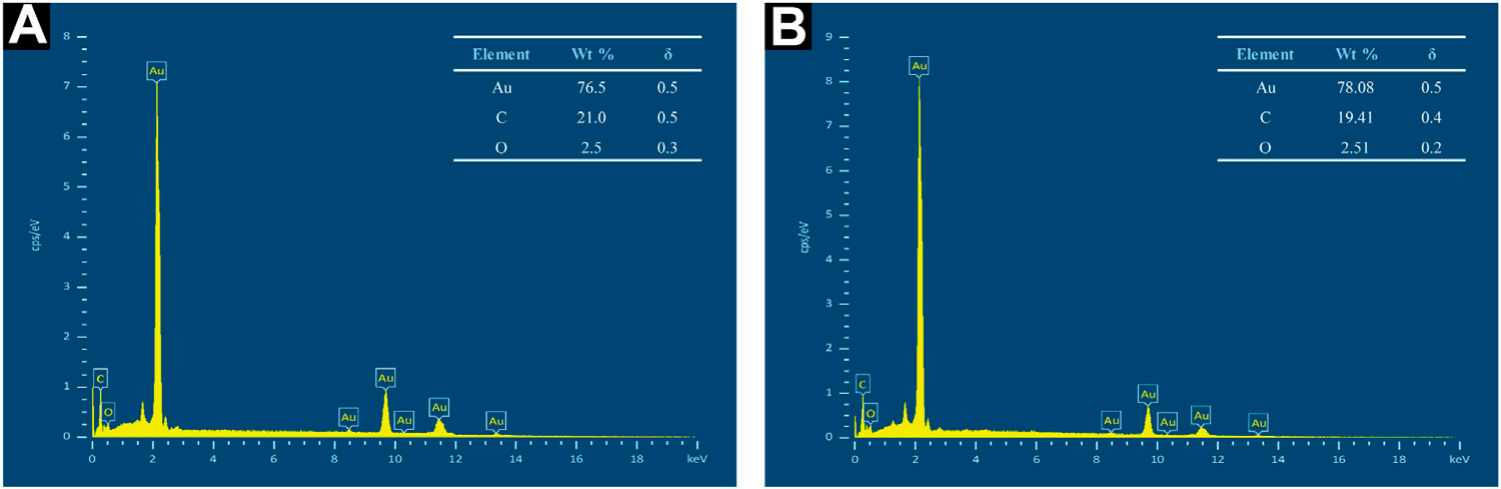
EDX spectra of **(A)** AuNPs@pH6 and **(B)** AuNPs@pH10

Henceforth, EDX measurement was performed to generate a semi-quantitative elemental analysis of Au atoms biosynthesized in this work. A strong and sharp peak was observed in the EDX spectra of both AuNP samples concurrent with the elemental Au composition, as seen in Figure 10A,B. Furthermore, weak carbon (C) and oxygen (O) peaks were observed, possibly attributable to X-ray emission from biomolecules capped on the Au atom surface or in the particle vicinity. The measurement of C element, however, might have included readings from the conductive double-sided C adhesive tape used to secure the pellets to the brass stubs. Overall, the spectra verified the elemental Au formed *via* aqueous CB extract incorporation.

### 3.6 Crystallographic Analysis

**FIGURE 11:**
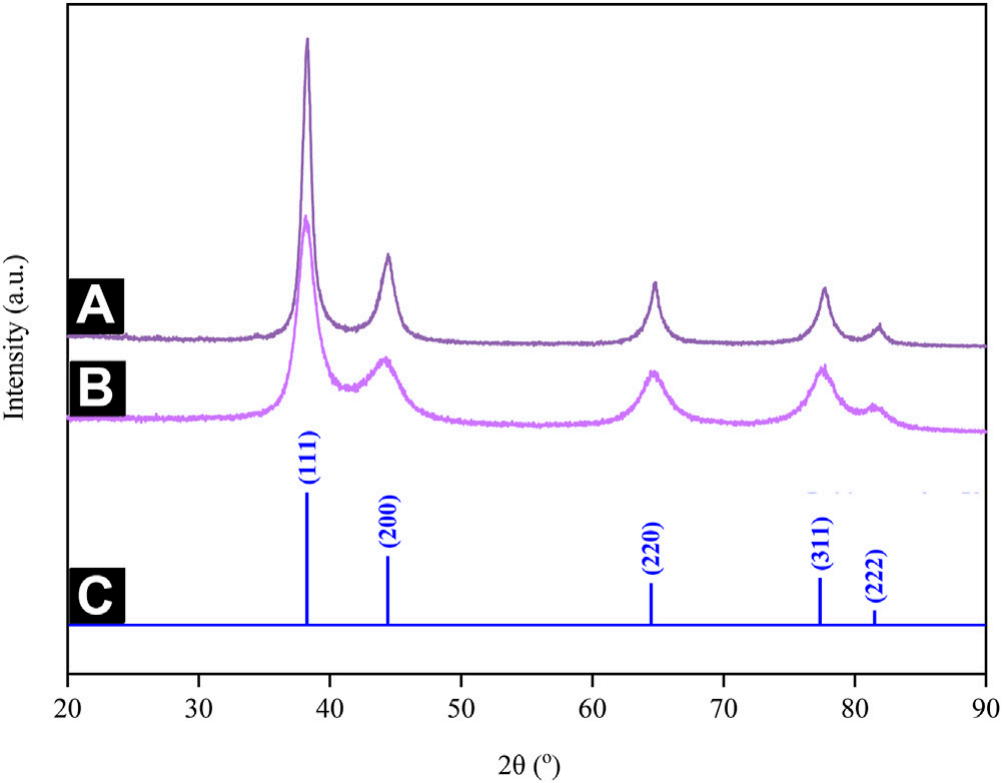
XRD patterns of **(A)** AuNPs@pH6 and **(B)** AuNPs@pH10 along with the **(C)** stick patterns for the JCPDS file no. 04-0,784.

This study employed XRD to ascertain the crystal structure of Au atoms biosynthesized by using CB extract. Figure 11A,B depict the XRD patterns generated, which pose five distinct sets of lattice planes indexed to the (111), (200), (220), (311), and (222) facets of the Au atoms. They confirmed that the Au atoms formed have a face-centered cubic (FCC) crystalline structure, which is in good agreement with the standard JCPDS 04-0784 (Figure 11C). Meanwhile, the diffractogram for AuNPs@pH10 depicted broadened peaks, implying a reduced crystallite size. Here, the crystallite sizes of AuNPs@pH6 and AuNPs@pH10 corresponding to the intense (111) plane are 10.45 and 2.83 nm, respectively, as calculated using Scherrer’s equation.

### 3.7 Functional Group Analysis

**FIGURE 12:**
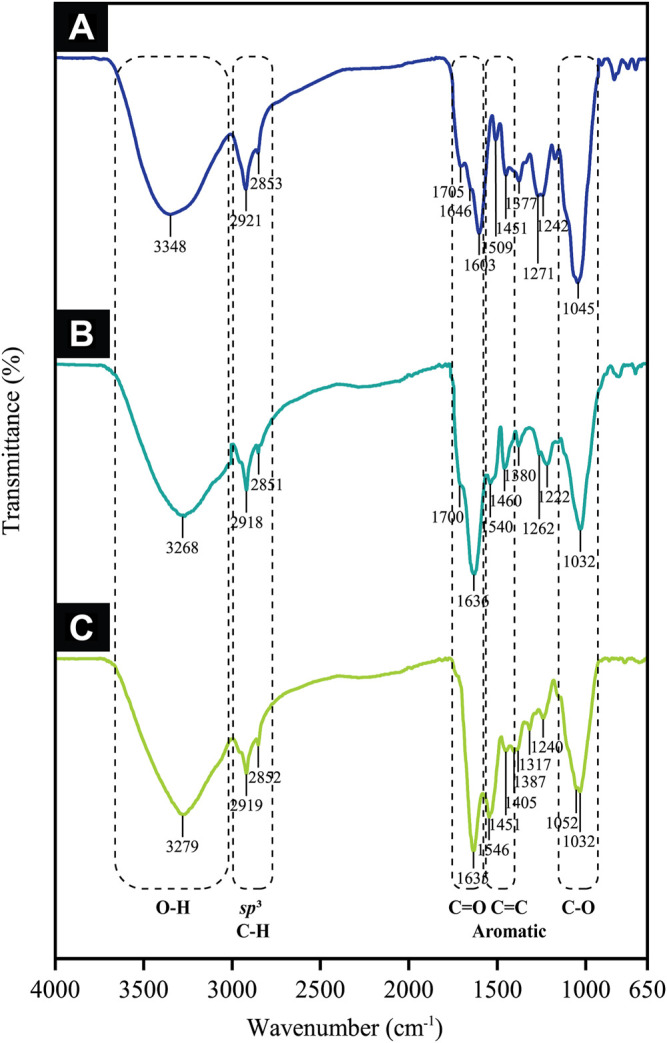
FTIR spectra of **(A)** CB extract, **(B)** AuNPs@pH6 and **(C)** AuNPs@pH10

This study distinguished the functional groups present in the CB extract for the reduction of Au ions to Au atoms by employing FTIR in which its spectra revealed five major peaks pre- and post-trivalent Au ion bio-reduction. This is depicted in Figure 12, whereby the characteristic hydroxyl group was identified at 3,348 cm^−1^ for the extract, which then shifted to 3,268 cm^−1^ in AuNPs@pH6 and to 3,279 cm^−1^ in AuNPs@pH10, thereby proposing its involvement in CB-AuNP bio-synthesis. Such outcome is excellently supported by the phytochemical screening results seen in Table 1, indicative of phenolic compounds and flavonoids prevalent as the secondary metabolites. Contrarily, peaks occurring at 2,919 and 2,851 cm^−1^ in the extract spectrum could be associated with the C–H stretching of alkanes, which did not shift much following CB-AuNP synthesis. Meanwhile, the two peaks at 1705 and 1,646 cm^−1^ in the CB corresponded to the C=O stretching vibrations, thereby implying the role of phenols/flavonoids. Their function was specifically in view of reducing Au^3+^ to AuNPs and undergoing complexation (or adsorption) onto the surface of the AuNPs. Both classes of compound are found here in the extract as per the phytochemical analyses, thus corroborating the FT-IR results. It should be noted that the weak to moderately intense peak observed at 1700 cm^−1^ in the AuNPs@pH6 spectrum suggested the role of carbonyl functional groups in Au ion reduction. Gangula et al. (2011) have detailed the possible binding of flavanones and terpenoids to the AuNP surface following their interaction through carbonyl groups or π electrons. Bands centered around 1,600, 1,500 and 1,450 cm^−1^ are associated with C=C aromatics, while signals at 1,052, 1,045 and 1,032 cm^−1^ identified with the C-O groups of polyphenols. Henceforth, the phytochemical tests and FT-IR results collectively imply the presence of phenolics and flavonoids in the extract is likely to impact the Au ion reduction and/or they are subjected to complexation (or adsorption) on the CB-AuNP surface.

### 3.8 Catalytic Reduction of 4-NP

**FIGURE 13:**
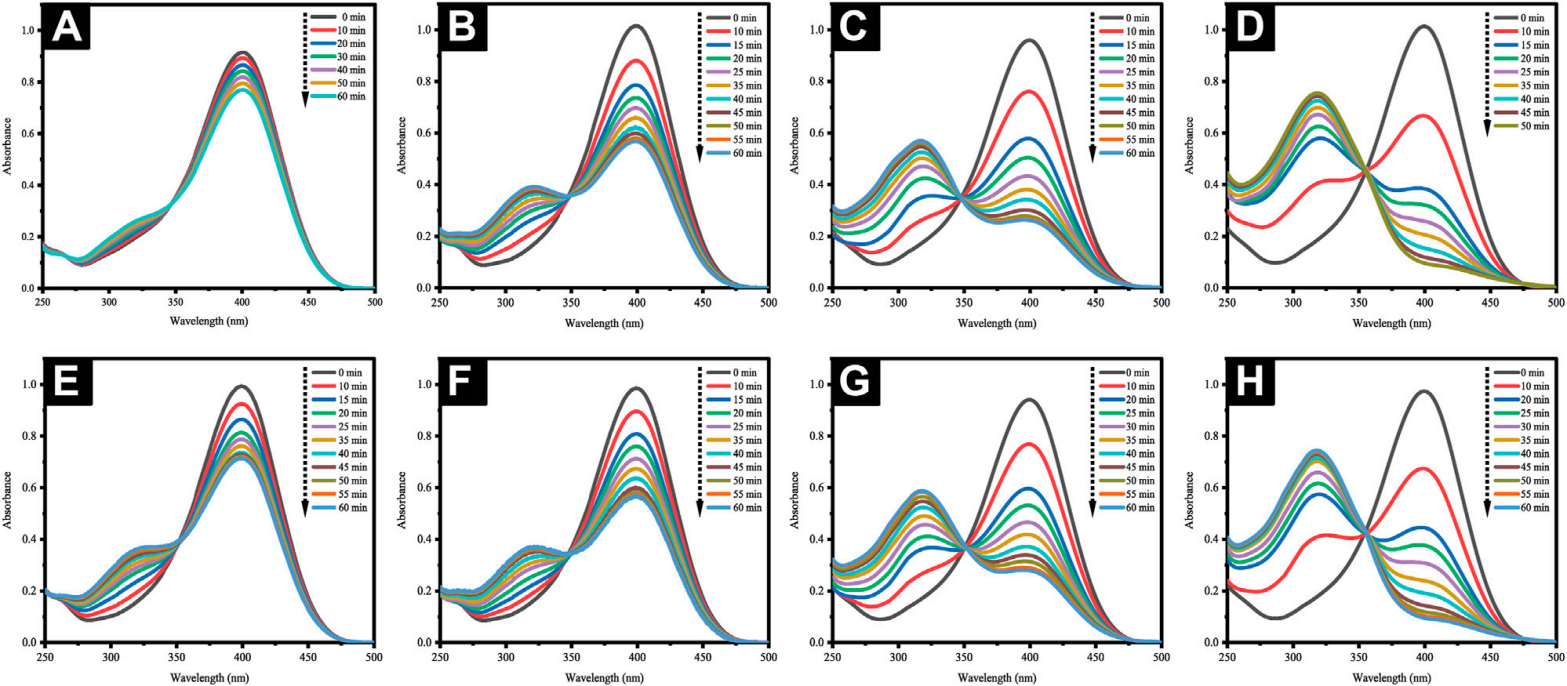
**(A)** UV-Vis spectra of 4-nitrophenolate ions devoid of AuNPs. Time-dependent UV-Vis spectra for the reduction of 4-NP with AuNPs@pH10: **(B)** 1 mg, **(C)** 2 mg and **(D)** 3 mg; AuNPs@pH6: **(E)** 1 mg, **(F)** 2 mg, **(G)** 3 mg and **(H)** 4 mg.

**FIGURE 14:**
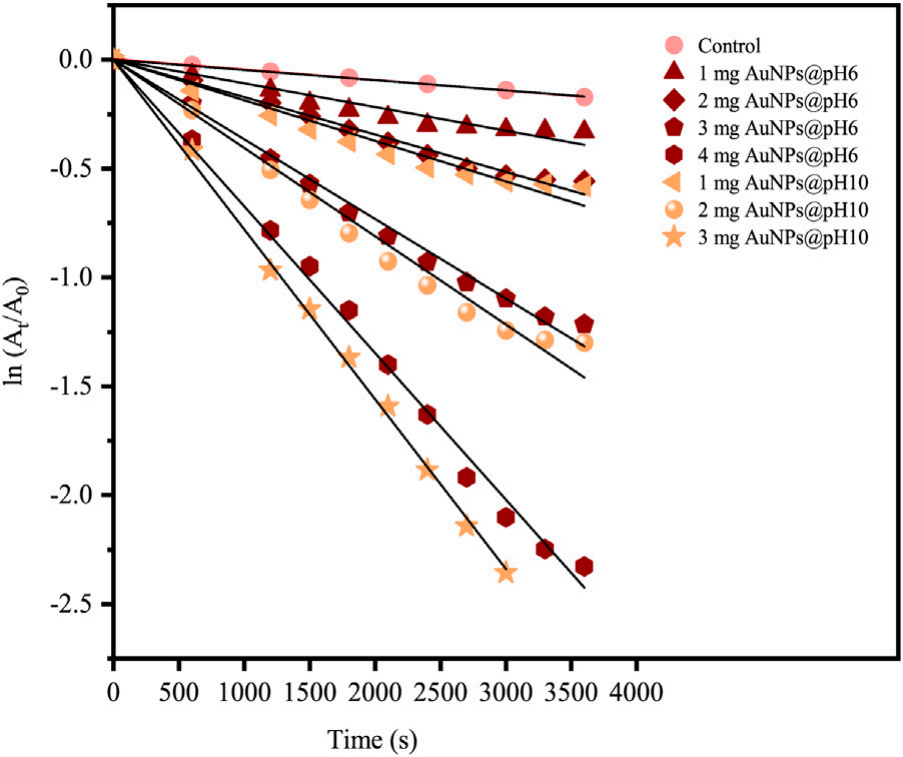
A plot of ln (A_t_/A_0_) versus time (s) for the reduction of 4-NP using biosynthesized CB-AuNPs.

Theoretically, the 4-NP reduction reaction is a common model reaction implemented in assessing the catalytic activity of AuNPs, which is especially preferred due to the ease in following the reaction progress by using UV-visible spectrophotometry. Here, an excessive three-fold amount of hydrazine hydrate relative to the 4-NP concentration was incorporated in ensuring pseudo-first-order kinetics. The presence of the former allowed the formation of 4-nitrophenolate ions absorbed at 400 nm. In the absence of AuNP catalysts, however, the abundance of 4-nitrophenolate ions would be unchanging (Figure 13A). Following the addition of either AuNPs@pH6 or AuNPs@pH10, the absorbance at 400 nm shows a decreasing trend, as seen in Figures 13B–G. An absorbance peak then materialized at 317 nm concomitant to the reaction product of 4-AP. In particular, Figures 13B–D reveal that the amount of AuNPs@pH10 impacts the time duration necessary for reaction completion; 1 mg for 44.1%, 2 mg for 72.7%, and 3 mg for 91.7%. For AuNPs@pH6, however, increments of the reaction rate also occur concurrently with the amount of the nanocatalyst as seen in Figures 13E–H, albeit the higher amount required for reaction completion compared to AuNPs@pH10. Here, Figure 14 details the correlation between ln (C_t_/C_0_) and time (s), where C_t_ and C_0_ denote the 4-NP concentrations at time t and 0, respectively. Accordingly, the linear relationship of AuNPs@pH6 and AuNPs@pH10 revealed the reaction to adhere to the pseudo-first-order kinetics. Meanwhile, the rate constant (k) is generated from the slope of the linear equation as follows:

$$-kt=In\frac{C_{t}}{C_{0}}=In\frac{A_{t}}{A_{0}}$$ (3)

where A_t_ and A_0_ are substituted for C_t_ and C_0_, respectively. A_t_ and A_0_ are the absorbances of 4-NP at 400 nm at time t and time 0, respectively.

Figure 14 reveals an increment of reaction rate along with the AuNPs@pH10 amount employed for the catalytic reduction reaction, which was also seen for AuNPs@pH6. Here, the rate constant of AuNPs@pH10 (8.24 × 10^−4^ s ^−1^) was slightly higher than that of AuNPs@pH6 (6.31 × 10^−4^ s ^−1^). The comparatively smaller sizes of AuNPs@pH10 may be one reason contributing to the faster reaction kinetics. In addition, the adsorption of oxidized phenols at the surface of green NPs, which suppresses the adsorption of nitroaromatic substrates, may result in AuNPs@pH6 having slower kinetics than their chemical counterparts (Bogireddy et al., 2018).

The obtained rate constants (k) are widely used to compare the performance of various catalysts (Seo et al., 2017; Cui et al., 2019; Doan et al., 2020; Zhang et al., 2020). Table 2 enlists several plant-mediated AuNP catalytic systems previously investigated for 4-NP degradation. In this work, however, the AuNPs demonstrated moderate catalytic activity in comparison to those shown in the literature. The performances of AuNPs@pH10 and AuNPs@pH6 were found to be almost incomparable to the isotropic and anisotropic AuNPs synthesized using *Coffee arabica* seeds at pH 10.5 and pH 5, respectively (Bogireddy et al., 2018). Moreover, AuNPs@pH6 had a higher reaction rate as opposed to anisotropic AuNPs that were synthesized biosynthetically using *Cochlospermum religiosum* roots (Maity et al., 2012) and *Salvia officianalis* leaves (Oueslati et al., 2020). Contrarily, ultra-small isotropic AuNPs produced using the stems of *Salvia officianalis* depicted notably higher catalytic activity (2.56 × 10^−3^ s^−1^) than that of the AuNPs@pH10. Additionally, the isotropic AuNPs@pH10 offered a catalytic performance similar of that shown by *Bupleurum falcatum* (Lee et al., 2015), but they are less effective compared to those synthesized using *Crinum latifolium* (Vo et al., 2019) *Lactuca indica*, (Vo et al., 2020), *Passiflora edulis* (Nguyen et al., 2021), and *Litsea cubeba* (Doan et al., 2020) extracts in prior works. Nonetheless, the current biosynthetic AuNPs proved their worth as a potential catalyst for the reduction of 4-NP.

**TABLE 2:** Comparative literature data of several plant-mediated AuNPs applying catalytic reduction of 4-NP.

Plant	Plant part(x)	Particle size (nm) and morphology	k (s^−1^)	References
*C. brachiata*	Leaves	8–13 (isotropic)	8.24 × 10^−4^	This work
		10–120 (anisotropic)	6.31 × 10^−4^	
*Coffee arabica*	Seeds	6–38 (isotropic)	8.7 × 10^−4^	Bogireddy et al. (2018)
		32–96 (anisotropic)	6.45 × 10^−4^	
*Salvia officianalis*	Stems	6 (isotropic)	2.56 × 10^−3^	Oueslati et al. (2020)
	Leaves	∼27 (anisotropic)	4.16 × 10^−4^	
*Cochlospermum religiosum*	Gum	6.9 (anisotropic)	4.45 × 10^−4^	Maity et al. (2012)
*Bupleurum falcatum*	Roots	8.2–12.8 (isotropic)	8.2 × 10^−4^	Lee et al. (2015)
*Crinum latifolium*	Leaves	17.6 (isotropic)	3.43 × 10^−3^	Vo et al. (2019)
*Lactuca indica*	Leaves	14.5 (isotropic)	1.3 × 10^−3^	Vo et al. (2020)
*Passiflora edulis*	Peels	7 (isotropic)	1.32 × 10^−3^	Nguyen et al. (2021)
*Litsea cubeba*	Fruits	8–18 (isotropic)	5.8 × 10^−3^	Doan et al. (2020)

**FIGURE 15:**
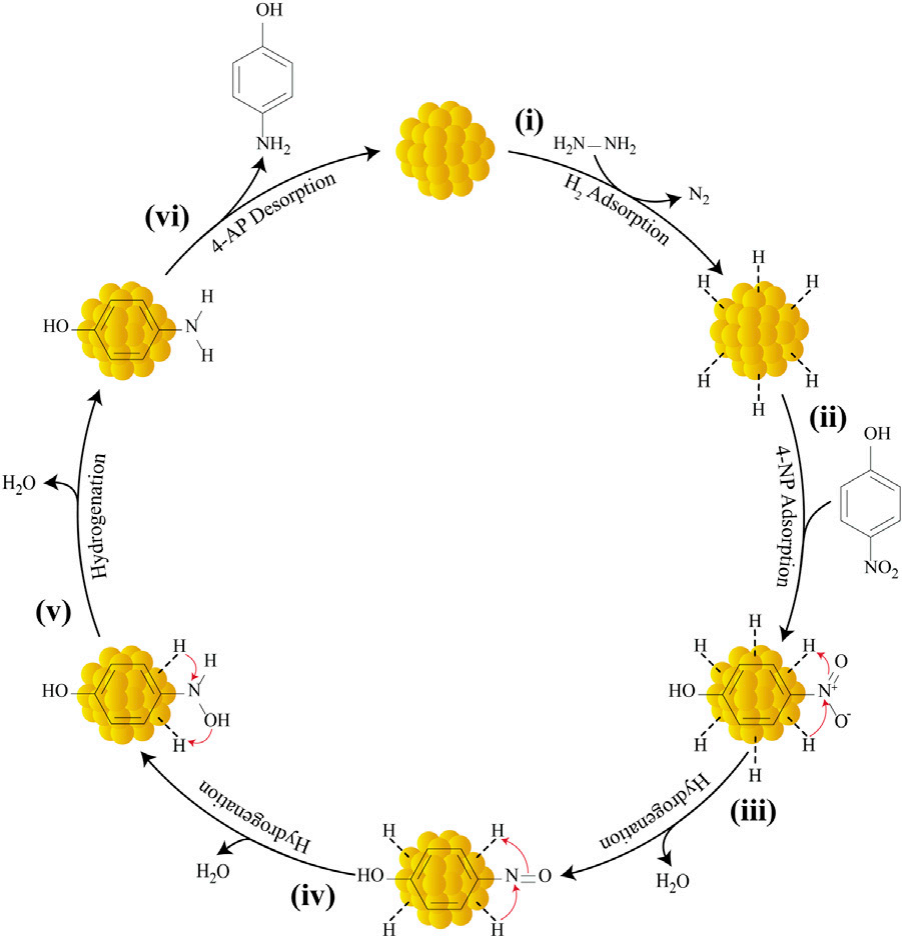
Mechanism for the catalytic reduction of 4-NP to 4-AP by AuNPs using hydrazine hydrate as the reducing agent.

With the help of several past studies, this work is able to delineate the mechanistic scenario for the reduction of 4-NP catalyzed by AuNPs using hydrazine hydrate as the hydrogen source (Blaser, 2006; Corma et al., 2007; Stratakis and Garcia, 2012; Gkizis et al., 2013; Datta et al., 2017). Generally, the process of converting 4-NP to 4-AP involves six main steps (Figure 15): 1) adsorption of reactant H_2_ molecules and 2) 4-NP onto the external AuNP surfaces; 3) nitrosophenol formation; 4) nitroso group conversion to hydroxylamine; 5) hydroxylamine conversion to 4-AP; and 6) 4-AP desorption. A total of six protons and six electrons (6H^+^, 6e^−^) are necessary for converting the nitro group (NO_2_) to an amino group (NH_2_). Reaction initiation occurs following hydrazine adsorption on the surface of AuNPs and bond dissociation, thereby producing nitrogen (N_2_) and surface-bound hydrogens as protons (H^+^) and hydrides (H^−^). The nitrophenols adsorb on the surface of AuNPs get transformed to nitrosophenol following H^+^ and H^−^ transfer, which is accompanied by dehydration process. Nucleophilic attack on these thermodynamically unstable nitroso moieties by H^−^ followed by protonation forms stable hydroxylamine; which is subsequently protonated and then attacked by H^−^, giving the desired 4-AP. Hydrogenation of hydroxylamine is a slow and rate-determining step. In the final step, the 4-AP are finally desorbed from the surface of the nanocatalyst to create a free space, allowing the reaction to continue.

## 4 Conclusion

This work depicted the implementation of natural *C. brachiata leaf* extract in producing shape and size-modulated crystalline AuNPs at room temperature, which was highly straight-forward, rapid, cost-effectual, and environmentally safe. In particular, the optimization studies proposed the critical role played by the pH extract in regulating the synthesis rate. Tetrachloroauric acid solution incubated with the CB extract at pH 6 successfully yielded multiple-shaped AuNPs (i.e., triangle, hexagon, pentagon, etc.) in a widely encompassing range of 10–120 nm. Meanwhile, the abundance of isotropic quasi-spherical NP growth depicting smaller sizes (8–13 nm) was prevalent upon using a basic aqueous extract. In this study, both bio-reduced AuNP samples revealed superior catalytic attributes for a 4-NP reduction reaction to yield 4-AP via hydrazine hydrate incorporation in the aqueous phase. Notably, our findings suggest that the fabricated AuNPs have size-dependent catalytic activity in the reduction of 4-NP with small, isotropic NPs exhibiting 1.33-fold higher catalytic activity than larger anisotropic structures. This equates to the larger surface area held by smaller particles which allows for more nitroaromatic substrates to be accommodated during the reduction process. In this regard, the current biosynthesis approach may be assessed in the future in more detail for identifying rapid organic synthesis and broad-ranging catalysis reactions.

## Data Availability

The original contributions presented in the study are included in the article/Supplementary Material, further inquiries can be directed to the corresponding author.
